# Treatment of hand and finger fractures with the Stryker Hand Plating System

**DOI:** 10.3205/000305

**Published:** 2022-03-31

**Authors:** Christoph Biehl, Sabine Stötzel, Lydia Schock, Gabor Szalay, Christian Heiss

**Affiliations:** 1 Department of Trauma, Hand and Reconstructive Surgery, University Hospital Gießen, Germany; 2 Experimental Trauma Surgery, Justus Liebig University of Gießen, Germany

**Keywords:** finger fracture, Stryker Plating System, osteosynthesis, metacarpal and phalanx fractures

## Abstract

**Objectives:** 10% of all fractures occur in the fingers and metacarpal region. Early mobilization with preservation of grip function is the goal of any therapy for these injuries. Osteosyntheses with plates are used in complex fractures that do not allow any other treatment. The aim of this retrospective study was to evaluate the performance and safety of the Stryker Hand System.

**Patients and methods:** Between 2010 and 2019, 190 patients underwent surgical treatment with plates for fractures of the fingers and metacarpal region. Of these, 140 operations could be analyzed according to the inclusion criteria based on clinical and radiological parameters.

**Results:** Three-quarters of the patients were male. The mean age at the time of surgery was 39.3±16 years. Falling was the leading cause for hand fractures, and the most common were fractures of the shaft (>52%). More than 15% were complex hand injuries with more than one fractured finger. The majority of patients were healthy non-smokers without systemic diseases and relevant medical history.

**Conclusion:** The Stryker Finger Plates are safe implants with good results that are consistent with those reported in the literature. The trend is also toward stable-angle implants for fracture treatment of the finger, in order to enable the earliest possible functional, safe mobilization.

**Level of Evidence:** Level: IV; outcome-study, retrospective

## Zusammenfassung

**Zielsetzung:** 10% aller Frakturen treten an Fingern und Mittelhand auf. Eine frühe Mobilisierung mit Erhalt der Greiffunktion ist das Ziel jeder Therapie dieser Verletzungen. Osteosynthesen mit Platten werden bei komplexen Frakturen eingesetzt, die eine andere Behandlung nicht zulassen. Das Ziel dieser retrospektiven Studie war es, die Leistungsfähigkeit und Sicherheit des Stryker-Hand-Systems zu evaluieren.

**Patienten und Methoden:** Zwischen 2010 und 2019 wurden 190 Patienten bei Frakturen im Bereich der Finger und Mittelhandknochen mit Platten operativ versorgt. Davon konnten 140 Operationen nach den Einschlusskriterien anhand von klinischen und radiologischen Parametern analysiert werden.

**Ergebnisse:** Drei Viertel der Patienten waren männlich. Das mittlere Alter zum Zeitpunkt der Operation betrug 39,3±15,97 Jahre. Stürze waren die häufigste Ursache für Handfrakturen, am häufigsten waren Schaftfrakturen (>52%). Mehr als 15% waren komplexe Handverletzungen mit mehr als einem gebrochenen Finger. Die Mehrzahl der Patienten waren gesunde Nichtraucher ohne systemische Erkrankungen und relevante Anamnese.

**Schlussfolgerung:** Die Stryker-Fingerplatten sind sichere Implantate mit guten Ergebnissen, die mit den in der Literatur berichteten Ergebnissen übereinstimmen. Der Trend geht auch bei der Frakturversorgung des Fingers zu winkelstabilen Implantaten, um eine frühestmögliche funktionelle, sichere Mobilisierung zu ermöglichen.

**Evidenzgrad:** IV; retrospektive Ergebnis-Studie

## Objectives

Hands and fingers, as exposed gripping organs of humans, are exposed to injuries. The metacarpals and fingers account for about 10% of all fractures. The vast majority of injuries to the fingers can be treated conservatively [1]. The indication for surgical treatment is always given when open or unstable fractures or intra-articular fractures or rotation defects are present [2]. Imaging is indispensable for confirming the diagnosis [3]. The aim of the operation is an early mobilization of the finger and the soft tissue close to the bone to preserve the gripping function [4]. In addition to the anatomical reconstruction of the bone, the therapy takes into account the protection of tendons and tendon gliding tissue to avoid unnecessary restrictions [5], [6], [7]. If using minimally invasive procedures like Kirschner wires (K-wires) or screws is not possible due to the fracture morphology, osteosyntheses with plate implants are used. In recent years, angle-stable implants have also been increasingly used in hand surgery to further improve early functional exercise. All procedures and therapists must be measured against the ‘restitutio ad integrum’.

The Stryker Hand Plating System is a comprehensive mini fragment fixation system of plates in varying lengths, shapes and widths (designs) consisting of two modules: the VariAx Hand Locking Plate, offering the benefit of variable angled locking, and the Profyle Hand Standard Plating, utilizing non-locking plates. The plating system offers locking and non-locking options necessary to treat a full range of hand (metacarpal) and wrist fractures. The system is intended for use in internal fixation of small bones of the hand (finger) and wrist. 

The objective of this retrospective study is to demonstrate the performance and safety of the Stryker Hand Plating System.

## Patients and methods

This retrospective study has been designed to collect safety and performance standard of care data on patients who have been previously operated (2010–2019) with the Stryker Hand Plating System for the treatment of hand and wrist fractures in adults (18 years and older) per indications for use. The study was approved by the institutional review board of the University Hospital Gießen and Marburg, Campus Gießen in Germany and was filed under the number AZ 45/19 in March 2019. First, bone consolidation in correct alignment, measured by radiographic and clinical assessments, documented the performance of the procedure. Second, the safety of the Stryker Hand Plating System was demonstrated through reporting of implant-related intraoperative and postoperative adverse events via a previously collected data set on the same sample population. Furthermore, pain or painlessness, as well as the ability to bear weight after consolidation were evaluated.

The authors included all patients who were operated on from 2010 to 2019 and met the protocol requirements. Data were collected as a consecutive case series from the investigator standard patient population. Patients with insufficient follow-up were documented but not included in analyses. Patients that met the following inclusion criteria were eligible for participation:

1. patients 18 years or older at the time of index procedure,
2. patients who previously received the Stryker Hand Plating System (VariAx and Profyle) by the indications for use: internal fixation of small bones including the hand and wrist.

Between 2010–2019, 190 patients were operated at the Department of Trauma, Hand and Reconstructive Surgery, using VariAx Hand Locking Plate Module or Profyle Hand Standard Plating Module systems. Eight adolescent patients were excluded from the study, because only adult patients were enrolled (see point 1. of the inclusion criteria). Furthermore, 42 patients without completely documented follow-up were excluded. In total, 140 patients participated in the study.

### Data collection (methods/sources)

The operation was performed on an outpatient basis, except for complex hand injuries. Data were collected at preoperative, operative/discharge, on the first postoperative day, after 3 and 6 weeks. At these points, an X-ray and a clinical examination were regularly performed. If the fracture did not consolidate, the period was extended by three weeks each time. From 3 months on, delayed fracture healing is defined, and from the 6^th^ month a pseudarthrosis. Metal removal was optional and was only done at device-related events or at the request of the patient.

Follow-up visits were performed until either bone consolidation had occurred, or the implant was removed (hardware removal) or otherwise determined to be a failure. Subjects for whom follow-up was insufficient to make this determination were documented, but not included in analyses.

### Methods of assessment/measurement

Only finger and metacarpal fractures were included in the study, and the fractures were classified into head, shaft, and base according to their location. Complex hand injuries were included in the same way. These are defined as multi-structural injuries of vessels, nerves, tendons, bones, and soft tissue that led to a restriction or loss of function.

Follow-up treatment was based on a checklist that included clinical evaluation for pain, exercise capacity, overall hand range of motion, and radiological control.

Only data on demographics, medical/surgical history, bone consolidation, and adverse events were included.

### Revisions/adverse events

Complications were classified as delayed bone healing, functional limitations, and severe complications such as deep infections.

### Statistics

All documented eligible patients operated between 2010 and 2019 were included. The table was screened for double-listed cases, and cases with missing follow-up consultations were excluded before statistical evaluation. This table is not provided in this report due to its scope and data protection.

A descriptive statistical evaluation was performed as mean ± standard deviation as well as the minimal and maximal values for age, body mass index, and consolidation time. Frequency analysis was performed for all other values (e.g. gender, race, medical history, injury and fracture classification) using Statistical Package PASW 26.0 (IBM, SPSS Inc., Armonk, NY, USA). Results were documented as absolute numbers and percentages (rounded to one decimal place)* of valid cases.

*Visualization was performed as pie charts. Because of the rounding to one decimal place, discrepancies from 100% at the first decimal place could occur.

## Results

### Study population

The patients of the study group were between 18 and 86 years of age (mean±SD: 39.3±16). The Body Mass Index (BMI) ranged between 19.2 and 40.6 (25.1±3.3). Almost three quarters of the patients were male (103 patients), with a minority of 37 female patients.

### Medical history

Standard medical history regarding smoking habits and other diseases revealed that 84 patients were non-smokers, nine were previous smokers, 41 were smokers, while six patients refrained from answering. Furthermore, only 7 patients had diabetes mellitus, 6 gave no information, and 127 were not diabetic. Only 5 patients had osteoporosis, another 5 gave no information and the remaining 130 patients were not osteoporotic. 33 of the patients included in this study had an additional medical history of interest, in one case this information was not available, and 106 patients had no additional relevant medical background.

The evaluated additional relevant medical history of the patients was manifold, including e.g. cardiac and lung diseases, psychological as well as cancer diseases, and drug abuse. 9 of the total number of 33 patients had more than one relevant additional medical condition. A complete list of the additional medical history documented during this study is listed in Table 1 (Tab. 1).

### Injury cause and type

**Figure 1:**
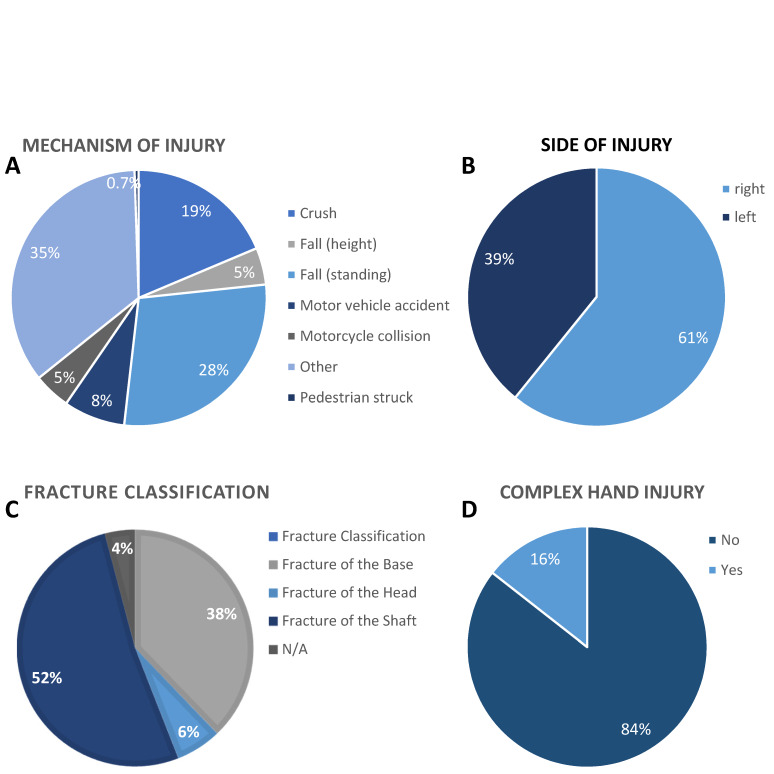
Falling was the leading cause for hand fractures and commonest were fractures of the shaft (metacarpal and finger). 13.5% of the hand fractures were caused by road accidents, 36.4% were the results of falls, 20.7% were caused by crushes and 29.3% of the hand fractures had other reasons (A). The right hand was the major (61.4%) side of injury (B). More than half of the fractures (52.1%) were classified as fractures of the shaft and fractures of the base (36.4%) were more common than fractures of the head (7.9%) and 3.6% of the fractures were not applicable (C). Complex hand injuries were identified in only 15.7% of the cases (D). All values are shown as percentage of 140 evaluated cases.

The initial accident that made the surgery necessary varied from road accident (10 cases of motor vehicle accidents, 8 cases of motorcycle collisions, and 1 pedestrian struck), falls (45 from standing and six from a height), 29 crushes, and 41 “other”, which were not defined in more detail (Figure 1A (Fig. 1)). In 86 cases the right hand was affected by the accident, and in 54 cases the left hand (Figure 1B (Fig. 1)). Fractures (metacarpal and finger fractures) were classified into “fracture of the base” (51 cases), “fracture of the shaft” (73 cases), “fracture of the head” (11 cases), and in 5 cases the classification was missing (Figure 1C (Fig. 1)). Fractures of the hand differ in severity and complexity. Therefore, the hand fractures in this study were classified as “complex hand injuries” in 22 of the evaluated 140 cases (Figure 1D (Fig. 1)).

### Primary indications and device use details

**Figure 2:**
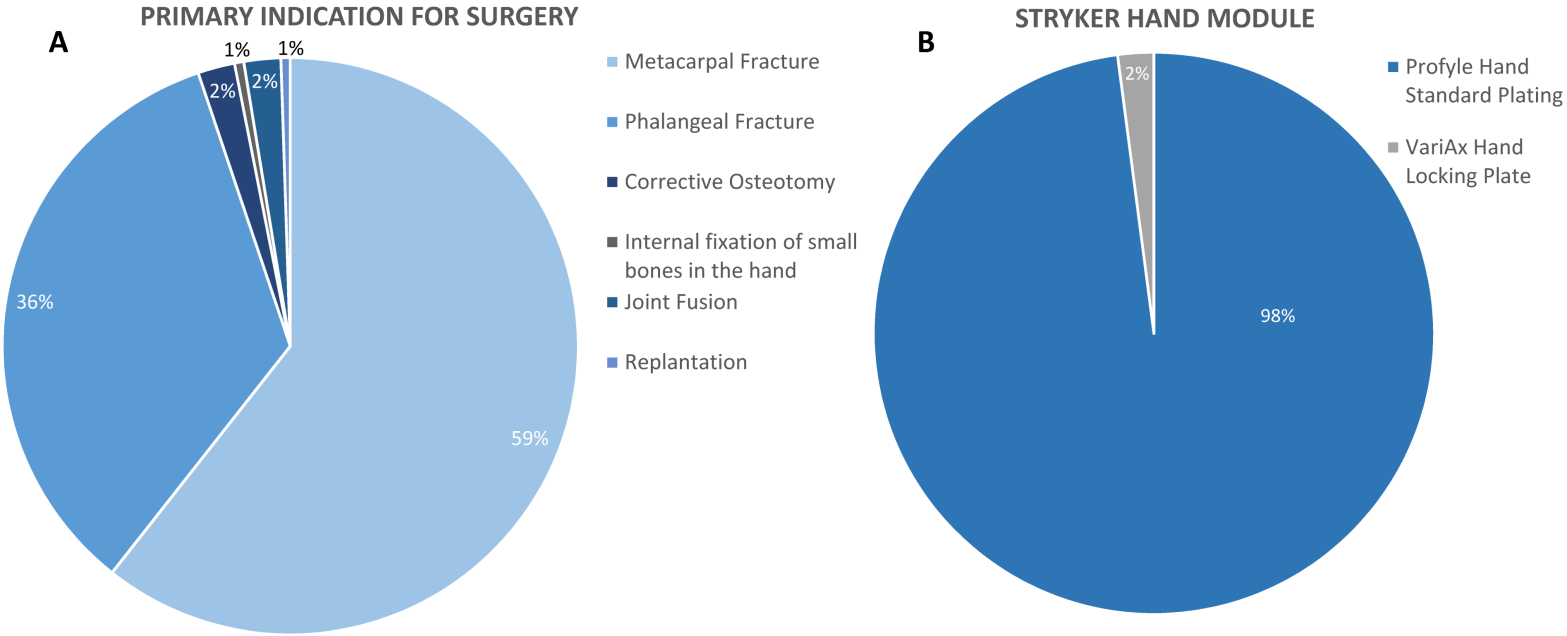
Non-locking plates were primarily used for surgical treatment of metacarpal and phalangeal fractures. Metacarpal (58.6%) and phalangeal (36.4%) were the most frequent fractures of the hand (A). Joint fusion was only represented with 2.9% and internal fixation of small bones in the hand, as well as replantation and corrective osteotomy each, were performed in only 0.7% of the cases. VariAx Hand Locking Plates were used in only 2.9% of the evaluated cases (B). All values are shown as percentage of 140 valid cases.

There were different indications that resulted in hand surgery (Figure 2A (Fig. 2)). In this study, the most common reasons were surgical treatment of metacarpal fractures (82 cases) and phalangeal fractures (52 cases). Joint fusion was the primary indication of surgery in 4 cases, one patient underwent hand surgery for internal fixation of small bones in the hand, one for corrective osteotomy, and another one for replantation. The Profyle Hand Standard Plating is a non-locking plate and was used in the majority (136) of the study cases, whereas the VariAx Hand Locking Plate was used in only 4 cases (Figure 2 (Fig. 2)).

### Bone consolidation (performance)

**Figure 3:**
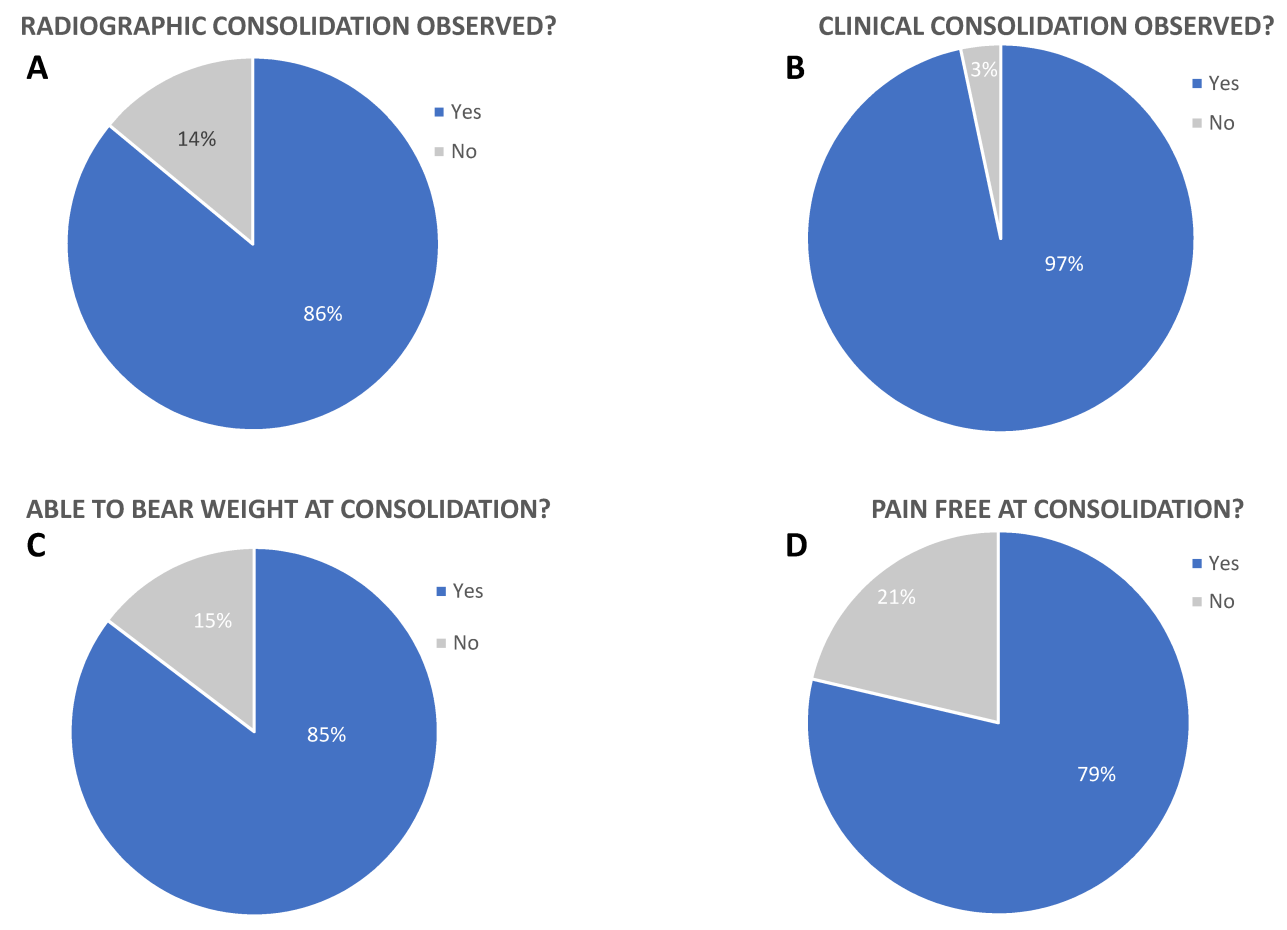
Primary endpoint of this study was defined as healing/performance of the implants. Consolidation was radiographically (A) and clinically (B) confirmed in 86.4% and 96.4% of all cases. 84.3% of patients were able to bear weight at consolidation (C) and 77.9% of all patients were pain free (D). All values are shown as percentage of 140 evaluated cases.

The major objective of surgical fracture treatment is regaining function. Therefore, bone consolidation was clinically and radiographically monitored, and the ability to bear weight as well as being pain-free were evaluated for all 140 evaluated cases (Figure 3 (Fig. 3)). X-rays were evaluated independently by radiologists and trauma surgeons using INFINITT PACS viewer, with joint discussion and evaluation of X-rays in difficult cases. No scoring system was used. Bone consolidation was observed radiographically in 121 cases after 93.2 days ((mean±SD)±55.7; min: 36; max: 212) (Table 2 (Tab. 2)).

In 19 fractures, no consolidation could be documented. The consolidation of the bone was also clinically evaluated. Hereby, 135 patients were positively rated, 5 cases showed no clinical consolidation, and only 2 of these 5 patients showed no clinical consolidation despite radiographically confirmed consolidation after 118 and 183 days, respectively.

The longest documented period was 1200 days for one patient who needed two revision surgeries to reach consolidation. Because of the revision surgeries, this case was excluded from the study.

3 patients showed neither radiographically nor clinically confirmed consolidation after 35 and 43 days, respectively. One patient developed a wound infection.

### Grip strength, movement and pain

118 of the patients in this study were able to grasp normally (radiologically and/or clinically determined) with the hand and fingers at consolidation, and 109 patients were pain-free at consolidation. Range of motion of the fractured finger was not differentially recorded. Only finger extension and fist closure were assessed, since the focus was on the use of the hand as a whole after surgical treatment. Only 22 patients were unable to use their hand at consolidation, and in 31 cases the goal of freedom from pain was not achieved (Figure 3 C–D (Fig. 3)).

### Device-related adverse events (implant safety)

Currently available implants for trauma and orthopedic surgery show a high biocompatibility. A removal of the implants after healing is mostly not planned and indicated. Therefore, a performed hardware removal as well as a delayed healing could be a sign of healing dysfunction or adverse events.

Device-related adverse events were seen in only 16 cases. 123 patients showed no signs of adverse events. For one patient, the information about adverse events was missing. The different types of adverse events and the number of cases in this study are listed in Table 3 (Tab. 3).

From the 140 patients included in the study, for 32 patients a delayed healing was documented, but 108 of the patients (77.1%) showed no signs of a delayed healing. Independent of the reason, a hardware removal was performed for 47 patients (33.6%), whereas the implants remained with the patients in 91 cases. The information about implant removal was not available in 2 cases.

A medical indication for the hardware removal was documented in 12 of the 47 cases of performed hardware removal. The device-related adverse events for the Profyle Hand Standard Plating were evaluated (Table 3 (Tab. 3)).

## Discussion

The aim of this retrospective study was to demonstrate the performance and safety of the Stryker Hand Plating System. This study differs from many publications that either compare different osteosynthesis procedures or focus on finger function [8], [9]. All patients of our hospital which underwent reconstruction of finger fractures with plate and with complete documentation were included.

The mean age of the patient population was 39 years. Compared with the collectives in the literature, the patients in our study are older, which may be country-specific. In comparison, the patients in the study by Egloff et al. were around 35 years old [10]. Pandey et al. report an average of 29 years in their Italian study [11], and Ahmed et al. have an average age in Pakistan of around 25 years [12]. An exception is the epidemiological study by Voth et al. on hand injuries in children [13].

75% of the patients requiring surgery in our collective were men. This is consistent with the patient collectives of Fusetti et al. [14].

In terms of medical history, age-adjusted data on tobacco use and concomitant diseases such as diabetes mellitus and osteoporosis correlate with the overall population of the country. In terms of surgical procedures and also potential complications, concomitant diseases seem to play only a minor role in the literature. The study group is too small for statistical conclusions about which comorbidities foster complications.

In this study, falls were the most common mechanism of injury leading to finger fractures. The dominant hand was involved in two thirds of the cases. As a national trauma center for occupational injuries, the proportion of occupational fractures and crush injuries at the hand was increased compared with the literature. Chung et al. identified falls as the main reason of a finger fracture in over 47% [15], and Voth et al. identified sports accidents in nearly 25% of under-18-year-olds [13]. No major differences were seen in the location of the fracture according to the literature. More than half of the surgically treated fractures are located in the shaft region, followed by fractures of the base. Fractures of the heads account for less than 10% of fractures at metacarpals and phalanges.

The use of plate systems for joint fusion or corrective osteotomies is reserved for individual cases after particularly strict indication. This is also consistent with reports in the literature [16]. The low proportion of angle-stable restorations in the collective is due to a late introduction of these systems in the hand. In the literature, improved early functional exercise is cited as an advantage of angle-stable plates [17], [18].

The primary indication for surgical treatment of metacarpal and phalanx fractures with a plate are dislocated, unstable fractures with rotational deformity and axial deviation [2]. Contrary to other authors, we do not consider the treatment of an open injury with plate osteosynthesis to be mandatory. In contrast, Haughton et al. say that “a fracture is best described as a soft tissue injury with an associated bony injury” [6]. Patient satisfaction with surgical treatment depends on good tendon function [19], [20]. Osteosynthesis should take this into account instead of additionally compromising the soft tissues by inserting (avoidable) plates [6], [21].

The consolidation time averaged 108.7 days, with the fastest consolidation after only 35 days, and the longest time period of 1200 days. In 85% of the fractures, consolidation was confirmed radiologically as well as clinically (119/140). In the literature, consolidation has been reported from 87% to 97% [12], [11], [22].

3 patients showed neither radiologically nor clinically confirmed consolidation at 35 and 43 days, respectively. One patient developed a wound infection.

### Revisions/adverse events

Complications must be distinguished between delayed bone healing, functional impairment, and severe complications such as deep infections. If the literature data on pseudarthrosis vary from 30–36%, the limitations of finger function are only about 10%. Limits of function are most severe for patients [23]. Deep infections are rare at around 1% despite the often difficult soft-tissue situation on the fingers [24], [25], [26]. A similar distribution is also reflected in our patient collective with 11.4%. 84.3% of this study’s patients were able to bear weight at consolidation (radiologically and/or clinically determined) and 77.9% of the patients were pain free at consolidation. Only 22 patients were not able to bear weight at consolidation, and in 31 cases the aim of being pain-free was not reached (Figure 3C–D (Fig. 3)). A delayed healing was documented in 22.9% of the cases. Chen et al. reported similarly poor results [27]. This highlights a problem with the general treatment of complex finger fractures. Plates have the highest stability, compared with wires, but imply greater soft tissue irritation from surgery [28], [29], [30]. More stable injuries often heal with minimal functional deficit. Since to date the undoubted superiority of a fitting has not been proven, it remains the individualized treatment decision of the surgeon [31].

### Limitations

The retrospective study has some limitations. A prospective study design would be more valid for the conclusions. Preoperative history and functional tests (e.g. DASH score) could be assessed. Furthermore, a control group (different plate systems, different surgical treatment) is missing. However, this is due to the structure of the hospital on the one hand, and fracture morphology and indication on the other.

## Conclusion

The Stryker Finger Plates are safe implants with good results that are consistent with those reported in the literature. The trend is also toward stable-angle implants for fracture treatment of the finger, in order to enable the earliest possible functional, safe mobilization.

## Abbreviations, Software and Implants

- BMI: Body Mass Index
- K-wires: Kirschner wires
- INFINITT PACS (INFINITT Europe GmbH, Frankfurt, Germany)
- Profyle Hand Standard Plating (Stryker-Leibinger, Kalamazoo, MI, USA)
- Statistical Package PASW 26.0 (IBM, SPSS Inc., Armonk, NY, USA)
- VariAx Hand Locking Plate (Stryker-Leibinger, Kalamazoo, MI, USA)

## Notes

### Ethical approval

Ethical approval was obtained by the Ethics Committee of the Justus Liebig University Gießen prior to the beginning of the study (AZ 45/19).

The raw data are available from the already completed routine diagnostics. There were no expected burdens on the patients. The data was evaluated anonymously and descriptively (inventory).

### Funding

The current research is an investigator-initiated Post-Market Clinical Follow-up (PMCF) funded by Stryker European Operations LTD Anngrove, IDA Business & Technology Park, Carrigtwohill, Co Cork, T45HX08, Ireland. Stryker financially supported the study with a total of 31,480 EUR. 25,400 EUR for a scientific researcher, 4,780 EUR overhead for the university, 1,300 EUR for the reports. The funding plays no role in the surgical treatment, collection, management, analysis, as well as data interpretation. Final reports and publications will be independent of the financial supporter.

### Authors’ contributions

- Study conception and design: GS, Stryker
- Acquisition of data: CB, CH, LS, GS
- Data monitoring and statistical analysis: CB, SS, CH, GS
- Analysis and interpretation of data: CB, SS, CH
- Drafting of the manuscript: CB, SS
- Critical revision: CH, GS, LS, CB, SS

### Acknowledgments

We acknowledge financial support by Stryker European Operations LTD. In addition, we are thankful for the support of Open Access Publishing of Justus Liebig University Gießen. We are also immensely grateful to Susann Hoefer and colleagues from Stryker Company for their comments on an earlier version of the manuscript, although any errors are our own and should not tarnish the reputation of these esteemed persons. We would like to thank Dr. Martin Heinrich, Matthias Lany, Dr. Matthias Mülke, Dr. Claudiu Oltenau, Melanie Schlägner, and Dr. Susanne Tilp for their support in collecting the data and performing analysis.

### Competing interests

The authors declare that they have no competing interests.

## Figures and Tables

**Table 1:**
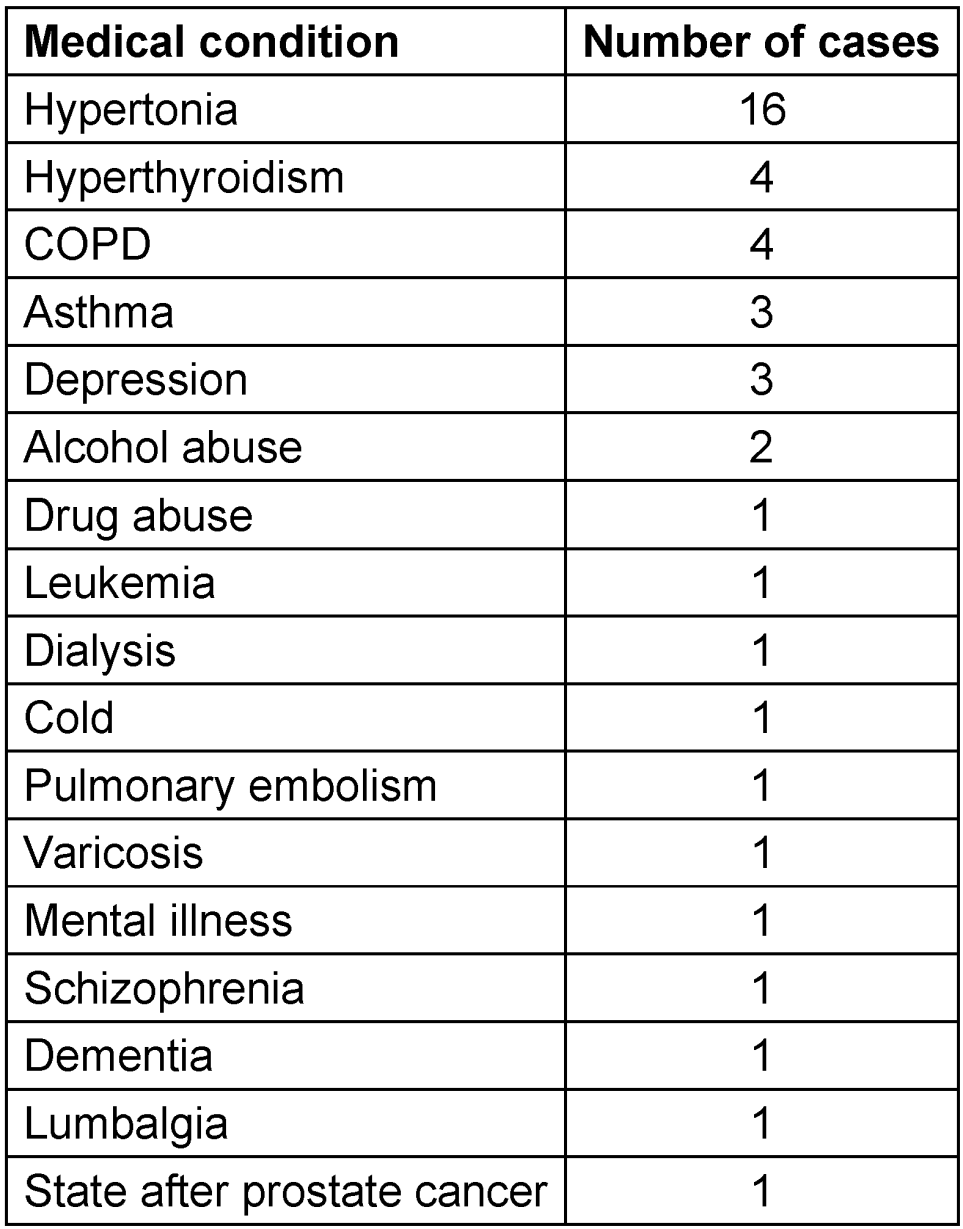
Additional relevant medical history of the study participants listed by diseases/medical condition and number of cases (N=33)

**Table 2:**
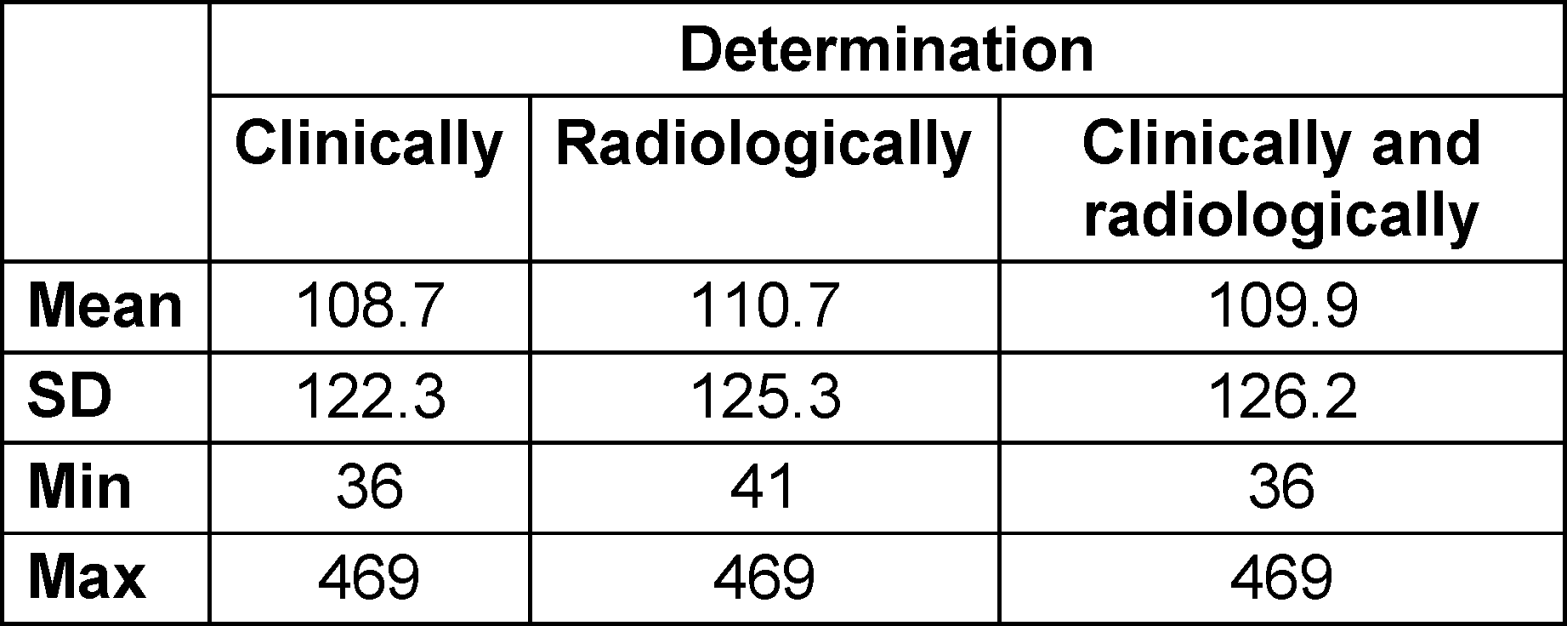
Bone consolidation in days

**Table 3:**
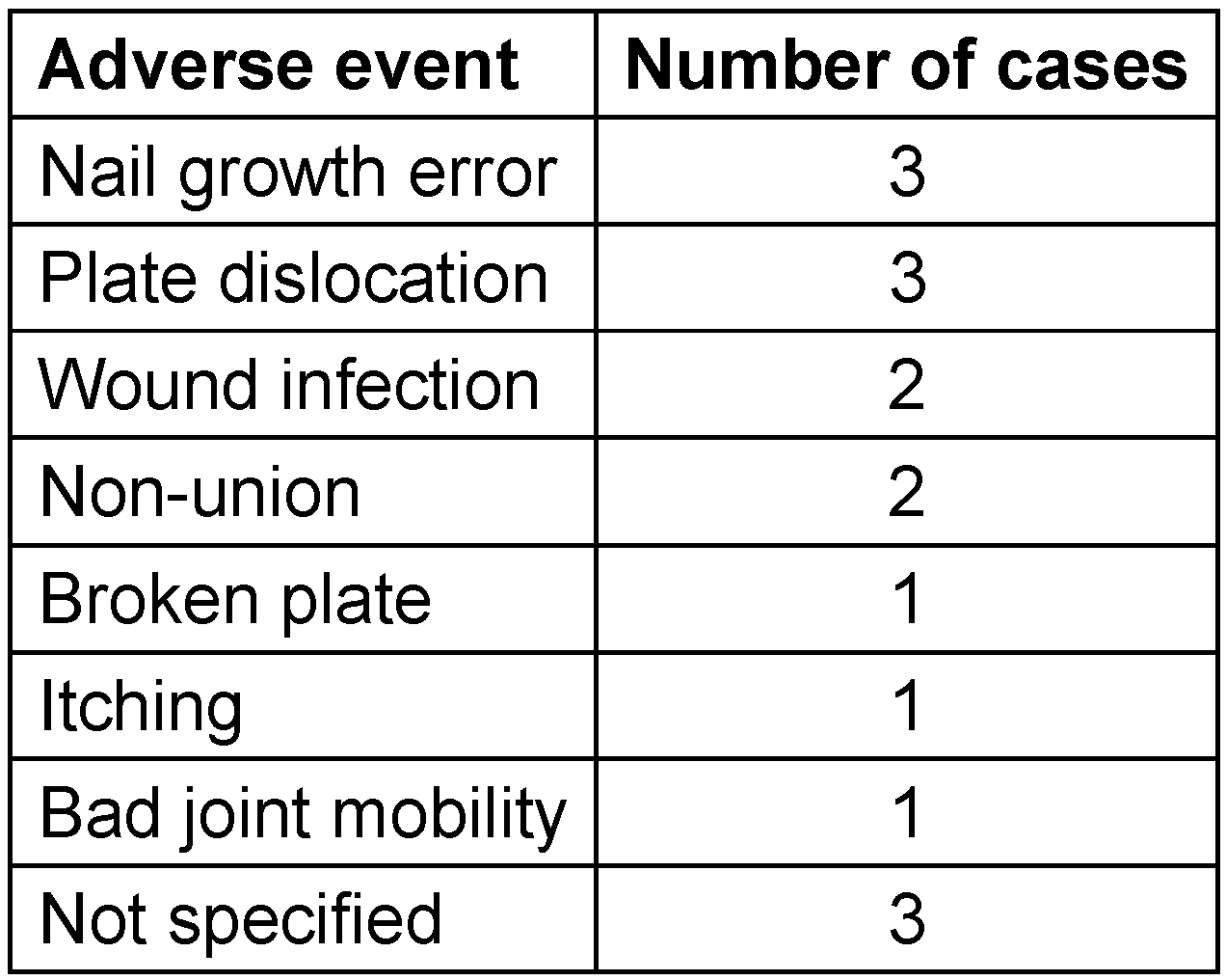
Observed adverse events are listed and the number of cases for each adverse event (total number of cases with adverse events = 16)
